# Clinical significance of stanniocalcin-1 detected in peripheral blood and bone marrow of esophageal squamous cell carcinoma patients

**DOI:** 10.1186/1756-9966-31-35

**Published:** 2012-04-26

**Authors:** Haizhu Song, Biao Xu, Jun Yi

**Affiliations:** 1 Department of Medical Oncology, Jinling Hospital, 305 East Zhongshan Road, Nanjing, 210002, P.R. China; 2 Department of Cardiothoracic Surgery, Jinling Hospital, 305 East Zhongshan Road, Nanjing, 210002, P.R. China

**Keywords:** Esophageal squamous cell carcinoma, Stanniocalcin-1, Disseminated tumor cells, Peripheral blood, Bone marrow, Micrometastasis, Prognosis

## Abstract

**Background:**

Stanniocalcin-1 (STC-1) is a potential marker of disseminated tumor cells (DTCs). The aim of this study was to examine STC-1 expression in peripheral blood (PB) and bone marrow (BM) of esophageal squamous cell carcinoma (ESCC) patients, and to evaluate its clinical significance.

**Methods:**

A total of 85 ESCC patients treated with radical resection were enrolled in this study. Immunohistochemistry was used to detect STC-1 protein expression in ESCC tissues. Nested RT-PCR was used to detect STC-1 mRNA expression in PB and BM.

**Results:**

There were 71 cases (83.5%) showed a higher level of STC-1 protein expression in tumor tissues than in adjacent normal tissues (*P* < 0.001). Furthermore, the frequencies of STC-1 mRNA expression detected in PB and BM were 37.6% (32/85) and 21.2% (18/85), respectively, and together increased sensitivity to 48.2% (41/85), which was much higher than that in patients with benign esophageal disease (5.0%, 2/40, *P* < 0.001). In addition, STC-1 mRNA expression either in PB or BM was correlated with lymph metastasis, advanced stage and adverse 2-year progression free survival (PFS). In a multivariate analysis using the Cox proportional hazard model, STC-1 expression in PB and/or BM was an independent unfavorable prognostic factor for ESCC, apart from lymph metastasis and clinical stage.

**Conclusions:**

STC-1 mRNA expression is a reliable marker for detection of DTCs in PB and BM of ESCC patients, and STC-1-positive DTCs may be a promising tool for diagnosis and prognosis assessment in ESCC.

## Background

Esophageal squamous cell carcinoma (ESCC) comprises the majority of esophageal cancer in China and it is characterized by a high incidence and mortality rate [1]. Even though this disease is surgically curable in the early stages, patients often suffer asymptomatic metastasis that is associated with a high mortality [2]. Evidences have shown that, cancer cells from the original region may disseminate into the peripheral blood (PB) or bone marrow (BM) in the early stage and survive without clinical representation as micrometastasis, an important initial step for recurrence and distant metastases [3,4]. Thus, it is clearly imperative to monitor these disseminated tumor cells (DTCs), which may contribute to improved diagnosis or prognosis and therefore more appropriate treatments.

As a result of the removal by immune system, very few DTCs exist and are undetected by normal methods. So far many different techniques have been applied for enriching and detecting DTCs, but the most commonly used is conventional reverse-transcriptase polymerase chain reaction (RT-PCR), because of the high degree of sensitivity and specificity, allowing the detection of one malignant cell among 10^6^ ~ 10^7^ monocytes [5]. Accordingly, an appropriate marker used in RT-PCR would be of a paramount importance, which should be expressed only in tumor cells, but not in normal cells. Previous studies have shown stanniocalcin-1 (STC-1) may be a promising tumor marker, for its high expression level in various of malignancies including ESCC, as compared with adjacent normal tissues [6-8]. Therefore, we determined the STC-1 mRNA expression using nested RT-PCR in PB and BM from ESCC patients treated with radical resection, and their associations with clinicopathological features and 2 year progression-free survival (PFS) were further evaluated.

## Methods

### Study population

This study enrolled 85 ESCC patients treated with radical resection at Jinling Hospital from July 2006 to July 2008. Patients consisted of 54 males and 31 females, with a median age of 62 (range, 44–83) years. Tumor stage was conducted according to the 7^th^ edition of the TNM staging system of the International Union Against Cancer [9], and patients were at stages I (n = 18), II (n = 25), III (n = 33) and IV(n = 9, supraclavicular or para-aortic lymph nodes metastasis). Cellular differentiation was graded according to the WHO grading system. Ethical approval was obtained from the hospital and informed consent was obtained from all patients prior to sample examination. Clinical follow-up data were available for all the patients. For each patient, 10 mL PB before surgery was collected, and PB mononuclear cells were isolated using Lymphocyte separation medium (Sigma, St. Louis, USA) according to the manufacturer’s protocol. Also, 5–10 mL of BM was aspirated from ribs during surgical treatment, and mononuclear cells were isolated from BM by Ficoll gradient centrifugation and then aliquoted to isolate RNA. PB and BM samples from 40 patients with benign esophageal disease were also collected.

### Immunohistochemical staining

Formalin-fixed, paraffin-embedded samples used for immunohistochemistry were sectioned at 2 μm thickness. Sections were deparaffinized using xylene, dehydrated by gradient ethanol, and then rehydrated with deionized water. Heat-mediated antigen retrieval was run by autoclave treatment (120°C for 2 min in 1 mmol/L ethylenediaminetetraacetic acid [EDTA], pH of 8.0) and then followed by cooling at room temperature. Incubation with a polyclonal goat anti-STC-1 antibody (diluted 1:200, Santa Cruz Biotechnology, CA, USA) was performed overnight at 4°C. After washing with phosphate-buffered saline (PBS), sections were then incubated with donkey anti-goat secondary antibody (Santa Cruz) for 30 min at room temperature. Coloration was performed with 3,3-diaminobenzidine. Nuclei were counterstained with hematoxylin. PBS was used as a negative control for the staining reactions. Immunostaining results were evaluated independently by 3 pathologists. The percentage of positive cells was rated as follows: 0 score for 0–5%, 1 score for 6–25%, 2 scores for 26–50%, and 3 scores for more than 50%. The staining intensity was rated as follows: 0 score for no staining, 1 score for weak staining, 2 scores for moderate staining, and 3 scores for strong staining [10]. The scores from the percentage and intensity were added to an overall score, and the expression of STC-1 protein in ESCC with an overall score of 0 was designated as ‘negative’, 1–2 was designated as ‘low’, 3–4 was designated as ‘moderate’ and 5–6 was designated as ‘high’.

### Nested RT-PCR

Total RNA in mononuclear cells was extracted by Trizol reagent (Invitrogen, Carlsbad, CA, USA) and RNA concentration was measured by spectrophotometer (BioPhotometer, Eppendorf, Hamburg, German). Approximately 1 μg of total RNA was used for cDNA synthesis using a PrimeScript™ 1st Strand cDNA Synthesis Kit (TaKaRa, Shiga, Japan). PCR reaction was performed in a 25 μl volume comprised of 5 μl of DNA template, 10 × Buffer, 0.15 mM dNTPs, 0.1 mM of each primer and 0.5U of Ex Taq Hot Start Version (Takara). Primer sequences and amplification conditions are listed in Table 1 and have been described elsewhere [11]. PCR products were identified on a 2% agarose gel containing ethidium bromide. A resected ESCC tumor tissue and water blank were used as positive and negative control, respectively.

**Table 1:** List of the nested PCR primers

Primers	Sequences (5′-3′)	Products	PCR conditions
STC-1 (outer)	CTTCACTCAAGCCAGGAGAGGGAAAGAGGAAA	890 bp	94°C for 30s, 62°C for 30s, 72°C for 1 min, 40 cycles
	TGGTGTGTCAACACCCCTAAAATGATA		
STC-1 (inner)	GTGGCGGCTCAAAACTCAGCTGAA	645 bp	94°C for 30s, 60°C for 30s, 72°C for 1 min, 40 cycles
	TTATGCACTCTCATGGGATGTGCGTT		
*β*-actin	CCCTGGACTTCGAGCAAGAGAT	531 bp	94°C for 30s, 55°C for 30s, 72°C for 1 min, 35 cycles
	GTTTTCTGCGCAAGTTAGG		

### Statistical analysis

Statistical tests were carried out using SPSS version 16.0 (SPSS Inc., Chicago, IL, USA). The differential expressions of STC-1 between tumor and adjacent normal specimens were calculated with Student’s t-test. Differences in frequency were assessed by Chi-square test or Fisher’s exact test. Overall survival curves were calculated using the Kaplan-Meier method and compared by log-rank testing. Multivariate Cox proportional hazard models were used to define the potential prognostic significance of individual parameter. *P* < 0.05 was taken as statistically significant.

## Results

### STC-1 protein expression profiles in ESCC tissue

**Figure 1:**
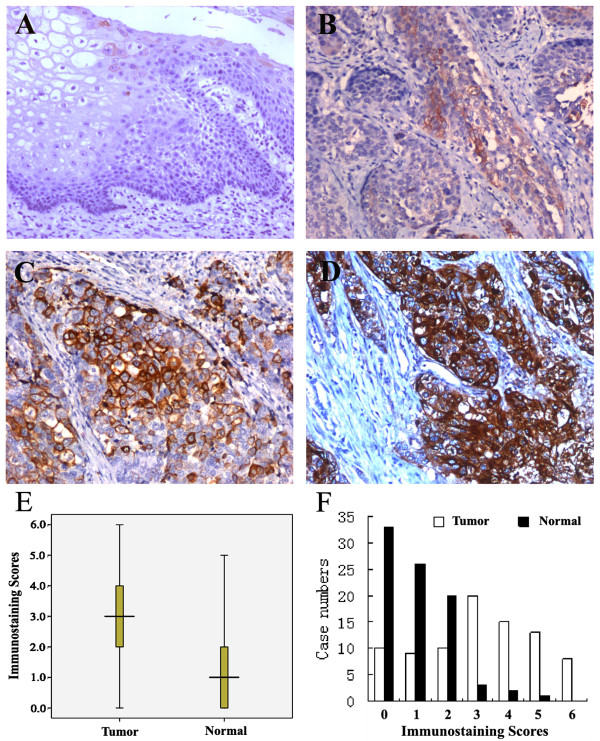
**STC-1 protein expression in ESCC and matched normal tissues determined by immunohistochemical staining (magnification × 200).** (**A**) STC-1 negative in normal tissue; (**B**) low STC-1 expression in tumor tissue; (**C**) moderate STC-1 expression in tumor tissue; (**D**) high STC-1 expression in tumor tissue. (**E**) The average immunostaining scores of STC-1 expression in tumor and normal tissues; (**F**) Distribution of immunostaining scores per sample in tumor and adjacent normal tissues.

We determined STC-1 protein expression in 85 pairs of ESCC and matched normal tissues by immunohistochemical staining. The representative immunohistochemical results are shown in Figure 1(A-D). In total, there were 71 cases (83.5%) showed a higher level of STC-1 protein expression in tumor tissues than in normal tissues, and the average immunostaining scores in tumor tissues were 3.08 ± 1.81 while in normal tissues was 1.05 ± 1.08 (Figure 1E, *P* < 0.001). Moreover, distribution of immunostaining scores per sample in tumor tissues and adjacent normal tissues was shown in Figure 1F, the rate of STC-1 protein high/moderate expression in ESCC and normal tissues was 65.9% (56/85) and 7.1% (6/85), respectively, which showed a significant difference (*P* < 0.001).

### STC-1 mRNA expression profiles in PB and BM from ESCC patients

**Figure 2:**
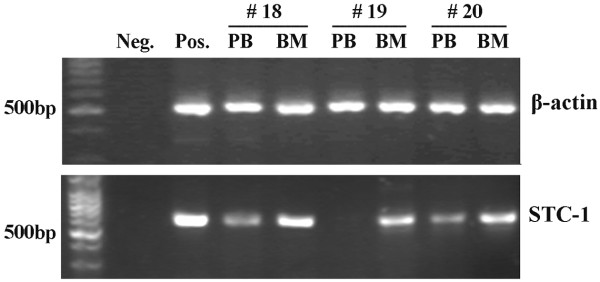
**Profiles of STC-1 mRNA expression in the peripheral blood (PB) and bone marrow (BM) of three ESCC patients.** Neg, a water blank was used as the negative control; Pos, a resected ESCC tumor tissue was used as the positive control.

The frequencies of STC-1 mRNA expression detected in PB and BM were 37.6% (32/85) and 21.2% (18/85), respectively, and showed no correlations with each other (*P* > 0.05), their combination increased the sensitivity to 48.2% (41/85) (Table 2). STC-1 mRNA detected in PB and/or BM was closely associated with its protein high/moderate expression in parallel tumor tissues, regardless of clinical staging (Table 3). Furthermore, in the 40 PB and/or BM samples from patients with benign esophageal disease, only 2 cases (5.0%) were found to be STC-1 mRNA-positive, this frequency was remarkably lower than that in the cancer patients (*P* < 0.001). Figure 2 shows the typical PCR results.

**Table 2:** STC-1 mRNA expression in peripheral blood and bone marrow of ESCC patients (n = 85)

peripheral blood	bone marrow		*P*-value
	STC-1 (+)	STC-1 (−)	
STC-1 (+)	9	23	0.223
STC-1 (−)	9	44	

(+), positive; (−), negative.

**Table 3:** Correlation of STC-1 expression in ESCC tissue and peripheral blood/bone marrow (n = 85)

Protein expression in ESCC tissue	peripheral blood /bone marrow		*P*-value
	STC-1 mRNA (+)	STC-1 mRNA (−)	
Stage I/II			
STC-1 high/moderate	11	11	0.012
STC-1 low/negative	3	18	
Stage III/IV			
STC-1 high/moderate	24	7	0.008
STC-1 low/negative	3	8	

(+), positive; (−), negative.

### Association between STC-1 mRNA expression and clinicopathological features

As shown in Table 4, STC-1 mRNA expression in PB and BM of ESCC patients were both associated with lymph metastasis and clinical stage. However, there were no correlations of STC-1 mRNA expression and patients’ gender, age, tumor site, depth and cellular differentiation.

**Table 4:** Association between STC-1 expression and clinicopathological features

Characteristics	No.	peripheral blood		bone marrow	
		STC-1 (+) (%)	*P*-value	STC-1 (+) (%)	*P*-value
Gender			0.674		0.429
Male	54	19(35.2%)		10(18.5%)	
Female	31	13(41.9%)		8 (25.8%)	
Age			0.242		0.446
<60	35	11 (31.4%)		6(17.1%)	
≥60	50	22 (44.0%)		12(24.0%)	
Tumor site			0.632		0.547
Upper thoracic	17	5 (29.4%)		4 (23.5%)	
Middle thoracic	33	12 (36.4%)		5 (15.2%)	
Lower thoracic	35	15 (42.9%)		9 (25.7%)	
Differentiation			0.615		0.575
Well	18	5 (27.8%)		3 (16.7%)	
Moderate	38	15(39.5%)		7 (18.4%)	
Poor	29	12(41.4%)		8 (27.6%)	
T status			0.583		0.329
T1 ~ 2	51	18 (35.3%)		9(17.6%)	
T3 ~ 4	34	14 (41.2%)		9(26.5%)	
Lymph metastasis			0.000*		0.013*
N_0_	41	7(17.1%)		4(9.76%)	
N_1_/N_2_/N_3_	44	25(56.8%)		14(31.8%)	
Clinical stage			0.020*		0.029*
I/II	43	11(25.6%)	23	5(11.6%)	20
III/IV	42	21(50.0%)	33	13(31.0%)	9

*P < 0.05.

### Association between STC-1 mRNA expression and ESCC prognosis

**Figure 3:**
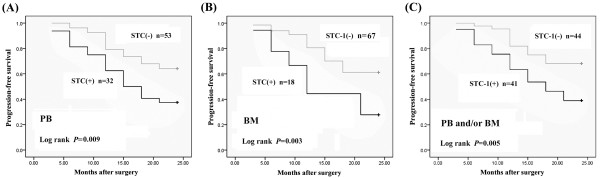
**Correlation between STC-1 mRNA expression in (A) peripheral blood (PB), (B) bone marrow (BM), and (C) PB and/or BM with 2-year progression-free survival among 85 ESCC patients using Kaplan-Meier statistical analyses.** (+), positive; (−), negative

To the follow-up deadline, there were 39 patients with progression or relapse within 2 years after the end of surgery. We performed univariate survival analyses to investigate the possible prognostic role of STC-1 expression in ESCC. As shown in Figure 3, the STC-1 expression in PB and BM were both associated with poor 2-year PFS (mean 16.2 months (95%CI: 13.688-18.750) *vs* 20.2 months (95%CI: 18.677-21.738), *P* = 0.009, and mean 15.0 months (95%CI: 11.543-18.457) *vs* 19.7 months (95%CI: 18.264-21.139), *P* = 0.003, respectively). Also in combination, patients with STC-1 mRNA expression in PB and/or BM showed a shortened PFS, as compared to that with STC-1 negative expression (mean 16.7 months (95%CI: 14.461-18.905) *vs* 20.6 months (95%CI: 19.014-22.167), *P* = 0.005).

Furthermore, multiple Cox regression analysis was used to verify whether the investigated variables including STC-1 expression were valid predictors of outcome after adjusting for potential confounding cofactors. Results showed that STC-1 expression in PB and/or BM, apart from lymph metastasis and advanced stage, were independent factors for predicting an adverse 2-year PFS for ESCC patients (Table 5).

**Table 5:** Multivariate analysis of clinicopathological factors for 2 year progression-free survival (PFS) of 85 patients with ESCC

Characteristics	Category	RR (95%CI)	*P-*value
Age	≥60 *vs* <60 years	1.500 (0.626-3.596)	0.363
Tumor differentiation	Poor *vs* Well/Moderate	1.607 (0.658-3.925)	0.296
T status	T3 ~ 4 *vs* T1 ~ 2	1.963 (0.814-4.733)	0.131
Lymph metastasis	N_1_/N_2_/N_3_*vs* N_0_	3.111 (1.276-7.583)	0.011*
Clinical stage	III/IV *vs* I/II	3.046 (1.255-7.395)	0.013*
STC-1 expression in PB and/or BM	Positive *vs* Negtive	3.348 (1.372-8.172)	0.007*
KPS scores	≥90 *vs* < 90	0.691 (0.281-1.703)	0.422

RR: Relative risk; PB: peripheral blood; BM: bone marrow; KPS: Karnofsky performance status.

*P < 0.05.

## Discussion

Hematogenous metastasis is the main cause of the poor outcomes for cancer patients, and there are many previous studies of DTCs that detach from the primary tumor, enter the bloodstream and travel via circulation to distant sites [12,13]. However, the relationships between BM micrometastases (BMM) and clinical outcome of ESCC are relatively insufficient [14]. BM is a major site for tumor cell deposition and dissemination. Evidences have shown that tumor cells spread into the BM while the primary tumor is still in the early stages, and BM acts as an intermediate site for target organ metastasis. Studies of BM samples by various methods have indicated that the presence or absence of BMM is associated with the clinical outcome of patients with esophageal carcinoma [15,16]. We currently investigated the DTCs in PB and BM by nested RT-PCR, to further confirm their clinical significance in ESCC.

Because PB and BM are mesenchymal tissues that do not normally express epithelial cell markers, detection of the expression of specific epithelial markers in the PB and BM implies the presence of metastatic cancer cells. Although many epithelial markers have been used previously, such as carcinoma embryonic antigen, cytokeratins and survivin, it is important to identify new potential biomarkers [14,15,17]. STC-1 is a kind of glycoprotein hormone, first found in bony fish and later in humans and mammals, with a highly conserved homology. Its primary function in fish is prevention of hypercalcemia and stimulation of phosphate reabsorption [18]. In mammals, STC-1 appears to play multiple roles in a series of biological processes, including pregnancy, lactation, angiogenesis, cerebral ischemia, oxidative stress and apoptosis [19-22]. Moreover, there is growing evidences suggesting that STC-1 is involved in carcinogenesis [23]. STC-1 expression levels are universally much higher in tumor tissues and cancer cell lines, such as hepatocellular, colorectal, ovarian, breast cancer and medullary thyroid cancer, than those in corresponding normal tissues [7,24-29]. Recently, Shirakawa *et al*[8] found that STC-1 mRNA and protein are overexpressed in ESCC tumors, compared with those in corresponding normal tissues, which significantly correlates with an advanced T status and poor prognosis for ESCC patients. This observation suggests that STC-1 may be useful as a tumor marker for ESCC. In fact, use of the STC-1 expression level as a diagnostic or prognostic biomarker in the blood has been validated in breast, lung, colorectal cancer, as well as hepatocellular carcinoma and leukemia [11,25,30-33]. The detection of STC-1 mRNA in BM has also been reported in breast cancer, which correlates with multiple histopathological prognostic factors, including primary tumor size, the number of positive lymph nodes and TNM stage [33].

In concordance with previous studies, we found that the level of STC-1 protein expression in ESCC was much higher than that in matched normal tissues, which further confirmed STC-1 as a promising tumor marker for ESCC. Moreover, STC-1 mRNA detection in PB and BM showed good sensitivity and specificity, the frequencies in PB and BM were 37.6% and 21.2%, respectively, which was comparable with other epithelial markers reported in ESCC. A previous study has indicated that DTCs detected in PB of breast cancer could not be an alternative to detect it in BM, because there are some different characters with each other [34]. We also found that DTCs detected in PB/BM had no correlations with each other, and together increased the sensitivity to 48.2%, which was much higher than that in controls with benign esophageal disease, and DTCs detected in PB and BM of ESCC patients were both associated with lymph metastasis, clinical stage and adverse prognosis. These results indicated that, DTC detection in PB is a non-invasive and more convenient method, but cannot replace that in BM, their combination will contribute to improve the test efficacy, and maybe useful as a diagnostic or prognositc biomarker.

Currently, the most important conventional prognostic factors for ESCC are the lesion length, invasion depth and lymph metastasis at the time of diagnosis (pTNM), which largely determines the treatment plan. However, the actual outcome of the disease is not entirely consistent with these clinicopathological parameters. Some patients at an early stage suffer tumor recurrence or metastasis soon after initial treatment, and others at advanced stages have long-term survival [35,36], which maybe due to the different molecular biology characteristics of their tumors, and DTC status may play an important role. A frequently updated pTNM still fails to discriminate between degrees of malignancy. Thus, in addition to these clinicopathological parameters, molecular markers are being sought in ESCC, and DTC dection has shown a promising prospect. Our study confirmed that DTC detected either in PB or BM of ESCC patients, which was represented by STC-1 mRNA expression, were both associated with an adverse 2 year PFS. These results were further verified with a Cox proportional hazard model, in which STC-1 mRNA expression in PB and/or BM from ESCC patients was found to be an independent unfavorable prognostic factor, apart from regional lymph metastasis and advanced stage. This suggests that DTC status may be a key factor determing the ESCC outcome. Thus, if a patient is found to be DTC-positive, comprehensive treatment including adjuvant radiochemotherapy should be recommended, which may improve patient survival by eliminating the DTCs and suppressing the micrometastasis.

## Conclusions

In this study, we performed nested RT-PCR to detect a potential representative biomarker of DTCs, STC-1 mRNA expression in PB and BM from ESCC patients. We found that STC-1 mRNA expression is a reliable marker to detect DTCs, and DTC positivity may be a promising indicator for diagnostic and prognostic assessment of ESCC.

## Abbreviations

BM = Bone marrow; DTCs = Disseminated tumor cells; ESCC = Esophageal squamous cell carcinoma; PB = Peripheral blood; PBMNCs = Peripheral blood mononuclear cells; PFS = Progression-free survival; RT-PCR = Reverse-transcriptase polymerase chain reaction; STC-1 = Stanniocalcin-1.

## Competing interests

The authors declare that they have no competing interests.

## Authors’ contributions

JY and HS designed the study. HS performed Nest RT-PCR. BX participated in the sample collection and performed the statistical analysis. HS drafted the manuscript. HS and JY revised the manuscript. All authors read and approved the final manuscript.
